# An Al-BERT-Bi-GRU-LDA algorithm for negative sentiment analysis on Bilibili comments

**DOI:** 10.7717/peerj-cs.2029

**Published:** 2024-05-15

**Authors:** Ziyu Liang, Jun Chen

**Affiliations:** School of Education, Guizhou Normal University, Guiyang, China

**Keywords:** Negative sentiment analysis, Fusion framework, Natural language processing, Online learning

## Abstract

The number of online self-learning users has been increasing due to the promotion of various lifelong learning programs. Unstructured commentary text related to their real learning experience regarding the learning process is generally published by users to show their opinions and complaints. The article aims to utilize the dataset of real text comments of 10 high school mathematics courses participated by high school students in the Bilibili platform and construct a hybrid algorithm called the Artificial Intelligence-Bidirectional Encoder Representations from Transformers (BERT) + Bidirectional Gated Recurrent Unit (BiGRU) and linear discriminant analysis (LDA) to crunch data and extract their sentiments. A series of tests regarding algorithm comparison were conducted on the educational review datasets. Comparative analysis found that the proposed algorithm achieves higher precision and lower loss rates than other alternative algorithms. Meanwhile, the loss ratio of the proposed algorithm was kept at a low level. At the topic mining level, the topic clustering of negative comments found that the barrage content was very messy and the complexity of the course content was generally reported by the students. Some problems related to videos were also mentioned. The outcomes are promising that the fundamental issues underlined by the students can be effectively resolved to improve curriculum and teaching quality.

## Introduction

In the era of digital learning, the learning methods utilized by students grow more diverse, not only for students in higher education but also for those in basic educational levels. So, the analysis method developed for online course platforms can be applied to MOOCs and other similar courses. For example, Chinese students often use a video-sharing website called Bilibili to learn and entertain. Many excellent first-line teachers and course developers have shared high-quality online course resources on the Bilibili platform. These online courses are free, open, and easy to use anytime. Since the online learning method requires users to be familiar with the technology to carry out regular educational and teaching activities, it is prone to new problems due to social reasons such as insufficient interaction between teachers and students, the lack of online learners’ emotional participation, and other issues.

On the other hand, online learners often communicate and interact in online discussion forums. These comments are complex and diverse regarding the content, cover a wide range of areas, and become the primary way to share and communicate among learners (Bai et al., 2019). In addition, the comments, for example, on the Bilibili platform are also very different from other types of comments, not only because the sentences are shorter but also because online learners will record their real experiences and feelings during the online learning in the comment area, which is related to the quality of courses, teachers’ performances and the service quality of the platform, which provides some inspiration and reference to improve the quality of courses.

When users’ sentiments are a concern, positive comments may be temporary, and the content of the comments may be monotonous. It may not contain substantial information that could not help improve the course content. At the same time, negative comments usually point out the shortcomings of courses and the service quality of the platform. Thus, teachers must pay close attention to negative comments (Li et al., 2019). However, teachers and course developers cannot read every negative comment pertinent to each course individually.

Moreover, micro texts are shorter and have a more diversified emotional vocabulary than short texts such as news, *etc*. Therefore, performing sentiment analysis on micro texts such as educational review texts is more challenging (Gui et al., 2017). The research employs hybrid sentiment classification algorithms to extract the emotional polarity of comment texts on online courses, primarily to derive negative texts with negative comments and carry out topic clustering, which helps assess online courses’ quality and provides helpful information for teachers. Hence, valuable feedback will promote the quality improvement of online courses.

The remainder of the article is organized as follows: Section ‘Related Works’ provides a literature review of sentiment analysis and its applications in education. Section ‘The Proposed Algorithm: ABGAL’ introduces the Al-BERT-BiGRU+LDA algorithm based on the attention mechanism. Section ‘Experimentation’ presents the experimental dataset and experimental results. Section ‘Conclusion’ concludes the research and presents a direction for future research.

## Related Works

### Pre-trained model

Most researchers (Szegedy et al., 2017; Wu, Shen & Van DenHengel, 2019; Singh, Hoiem & Forsyth, 2016) have confirmed through experimental studies that using pretraining technology can significantly improve the effect of downstream tasks. The early pretraining technique was a static encoding technique. In 2003, Bengio incorporated the idea of deep learning into the language model and proposed the NNLM (Bengio, Ducharme & Vincent, 2000) for a pretraining model.

In 2013, Word2Vec borrowed the idea from NNLM and employed a language model to construct word vectors. This was followed by Global Vectors (GloVe) (Pennington, Socher & Manning, 2014) and FastText (Joulin et al., 2016). Word2Vec and GloVe are the most widely used language models in natural language processing (NLP). Many studies have shown (Al-Amin, Islam & Uzzal, 2017; Tang et al., 2015; Grover & Leskovec, 2016) that the Word2Vec and GloVe models can significantly improve text vectorization’s representation effect in NLP tasks. Therefore, the vectorized representation of a text is fixed, and the model cannot effectively combine the text context information of the context globally. To resolve the problem of polysemy and word ambiguity and capture more useful deep semantic information, In 2018, ELMo (Sarzynska-Wawer et al., 2021) was proposed as a text representation method that could combine context. Since then, the GPT model (Radford et al., 2018), BERT algorithm (Devlin et al., 2018), *etc.*, have been suggested.

Moreover, it outperforms other pre-trained language algorithms in many typical tasks in NLP, which becomes an essential milestone in NLP. Our work uses the Al-BERT algorithm as the basic pretraining model. It obtains rich dynamic encoded semantic information through the Al-BERT algorithm to pave the way for deep text sentiment classification to extract text features better.

### Sentiment classification approaches

#### Traditional approaches for sentiment classification

First, the sentiment lexicon has been broadly implemented as an essential approach in sentiment classification tasks. Wilson, Wiebe & Hoffmann (2005) employed manual annotators to manually annotate a set of words to build a sentiment dictionary The algorithm can automatically detect the polarity of sentiment expressions in subsets and achieves excellent results. Tang et al. (2014) proposed an algorithm for constructing a large-scale emotional vocabulary based on representation learning methods. They introduced the Urban Dictionary to expand several emotional seed words to obtain more training data for constructing phrase-level emotional classifiers (Tang et al., 2014). Khoo & Johnkhan (2018) introduced a new universal sentiment dictionary, the WKWSCI sentiment dictionary, and conducted a comparative experiment with five existing sentiment dictionaries (HuLiu Opinion Lexicon, *etc*.) and found that Hu-Liu Opinion Lexicon performed well. It is suitable for sentiment mining of commentary texts, while the WKWSCI sentiment dictionary is suitable for non-commentary texts. Researchers have also tried combining sentiment lexicons with deep learning algorithms to improve classification performance in recent years. Yang et al. (2020) suggested a hybrid algorithm based on a sentiment lexicon combined with CNN and Bi-GRU based on an attention mechanism. Experimental results show that the algorithm can effectively improve the text performance of sentiment classification. However, due to the inability of the method used to construct the sentiment dictionary to unify contextual semantic features well, the classification precision of the algorithm is low, and there are issues such as an extended model construction period and slow update speed.

As classic supervised algorithms, machine learning algorithms usually implement sentiment classification through artificial feature engineering and achieve reliable classification results (Bao et al., 2021). Joachims (2005) suggested a new method to extract the emotional polarity of the text, that is, first determine whether a sentence expression is neutral or emotionally polarized and then distinguish the sentence expression with emotional polarity. Through this method, the emotional polarity of many sentence texts can be automatically identified. Classifying texts with negative reviews and implementing machine learning models also achieved good outcomes (Liu & Yang, 2020). Machine learning is representative of supervised learning. Model training must depend on much training data with manual annotations. Experts need to formulate rules and perform manual annotations in advance. Nevertheless, the process is difficult, and there are problems of poor portability and time-consuming and labor-intensive problems. Thus, a semi-supervised learning algorithm is an effective solution (Tan et al., 2021).

#### Sentiment classification based on deep learning approach

The last decade has witnessed deep learning gradually replacing machine learning algorithms, especially in NLP tasks, and its effects have been praised (Sailunaz & Alhajj, 2019; Xu et al., 2020; Kastrati, Imran & Kurti, 2020; Hassan & Mahmood, 2017; Chen et al., 2017). As a compelling model, CNN has made significant image and video processing breakthroughs due to its excellent local feature extraction ability. However, the learning ability for sequential data, especially text data, has been severely challenged (Liu & Guo, 2019). With the advent of recurrent neural networks (RNNs), Possibilities were seen for sequence data processing and prediction. RNN realizes the natural processing of time series data by storing and recalling information. However, in actual operation, we found that when RNN processed long text sequences, it was hard to converge during model training, impacting the training effect and model stability (Pascanu, Mikolov & Bengio, 2013). To resolve the issue, researchers proposed the LSTM algorithm (Hochreiter & Schmidhuber, 1997). LSTM preserved long-term dependencies on text content by introducing forget and update gates to manipulate, update, and ignore information in the hidden state. However, LSTM must deal with a long training time, numerous parameters, and high computational complexity. This complexity is the price of performance improvement, but it also becomes an obstacle to its use. Based on these issues, researchers combined linear variables with nonlinear gating structures to propose a more efficient GRU algorithm (Chung et al., 2014). In the design of GRU, by combining the update gate and the forget gate into one update gate, the network structure is simplified, parameters are reduced, calculations are saved, and better convergence is obtained. In addition, to get the front and rear information of the text sequence simultaneously, Bi-GRU integrates the forward and backward hidden layer information to attain richer contextual text information. This algorithm simplifies the model by unifying the update gate and the forget gate, which resolves the problem that RNNs face.

With the continuous advancement of deep learning algorithms, the attention mechanism has become widely implemented in various deep learning fields. Some researchers have applied it to text extraction and summary generation tasks. Rush, Chopra & Weston (2015) proposed an entirely data-driven approach to text summarization by employing a local attention model. Compared with other algorithms, the correct rate of summaries generated by the proposed method is greatly improved. Parikh et al. (2016) proposed a simple reasoning framework for NLP with the help of an attention mechanism, which can decompose complex text language into several simple subtexts. It dramatically reduces the complexity when a text is processed.

To sum up, LSTM, GRU, and Bi-GRU resolve a series of complex challenges that conventional algorithms face, especially in processing complex and extended sequences of text information. They show excellent performance and scalability, opened a new chapter in good model universality, and contributed significantly to improving and optimizing algorithms and effects in NLP. Therefore, this study combines the Al-BERT dynamic pretraining model with the Bi-GRU-Attention deep learning algorithm and the LDA model to obtain the deep emotional features of the learners’ comment texts and perform topic clustering and analysis on the negative comment texts.

## The proposed algorithm: ABGAL

**Figure 1:**
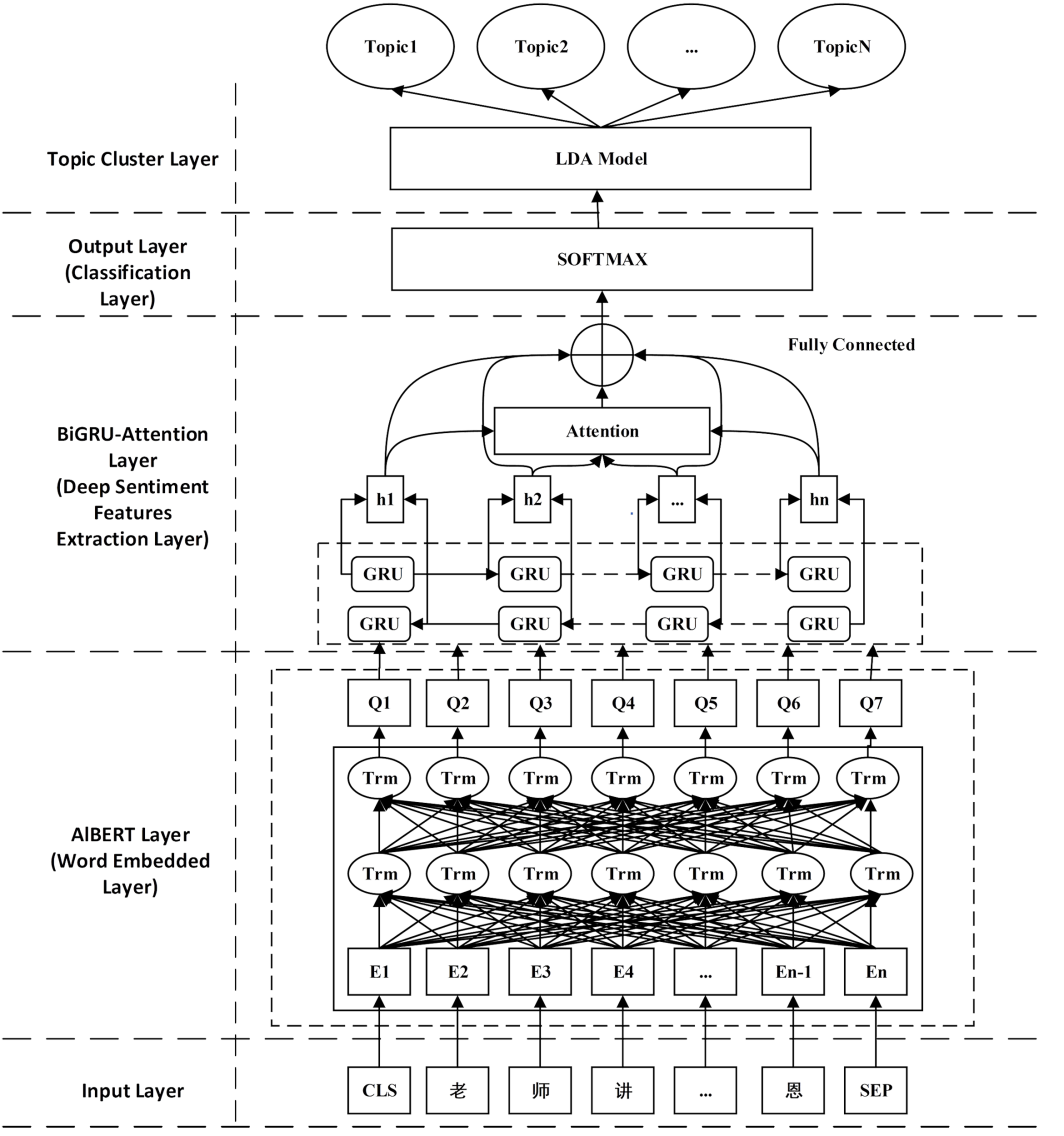
Structural diagram of ABGAL model.

This section introduces the algorithm architecture ABGAL. The model is constructed with two modules: the sentiment classification and the topic clustering. The sentiment classification module is divided into three layers: the Senti-Al-BERT layer, the deep Senti-extraction layer, and the softmax layer, respectively. After the sentiment classification module is run, the topic clustering module is built to realize the text topic clustering of negative comment texts to capture high-value information in negative comments. The diagram of the proposed algorithm is shown in Fig. 1.

### Al-BERT layer

The BERT model was suggested by Google in 2018 and achieved good results by stacking the Transformer substructure as a feature extractor and implementing the self-attention mechanism to encode words directly. However, when the model is expanded to a particular scale, the model training will take up excellent memory usage, the training time will be so long, and the model will degenerate and cause overfitting problems. To resolve these issues, scholars such as Lan et al. (2019) proposed a lightweight version of the BERT model, namely, Al-BERT (A Lite BERT), by employing the factorized embedding parameterization method and the cross-layer parameter sharing approach to cause the amount of the model’s parameter significantly reduced. It performs better on NLP tasks while reducing the number of parameters. Therefore, this study uses Al-BERT as a pretraining model. In the vectorized representation of text generated by Al-BERT, the vector corresponding to input in the algorithm has three superimposed vectors. It is called the Token Embedding Segment.

Embedding and Position Embedding. By converting each word into matrix–vector forms of Q, K, and V through different linear transformations and calculating independently of each other, a unique vector encoding for each term is obtained so that it can have a deeper perception of the context (Liu et al., 2020). Equation (1) presents the self-attention calculation.

(1) \begin{eqnarray*}\text{Attention}(Q,K,V)=\text{softmax} \left( \frac{Q{K}^{T}}{\sqrt{{d}_{k}}} \right) \cdot V\end{eqnarray*}

Q, K, and V denote the query, key, and value matrix, respectively. The research implements the trained Al-BERT algorithm, which consists of 12 layers of Transformer. The embedding dimension of the word vector is assigned to 768. To ensure that the vector input to the Al-BERT algorithm is easy to operate, this research sets the length of the input sequence to 512. Only the part within the maximum range is reserved for sentences exceeding the maximum length, and < padding > is used to fill in the insufficient length sequence.

### Bi-GRU layer

**Figure 2:**
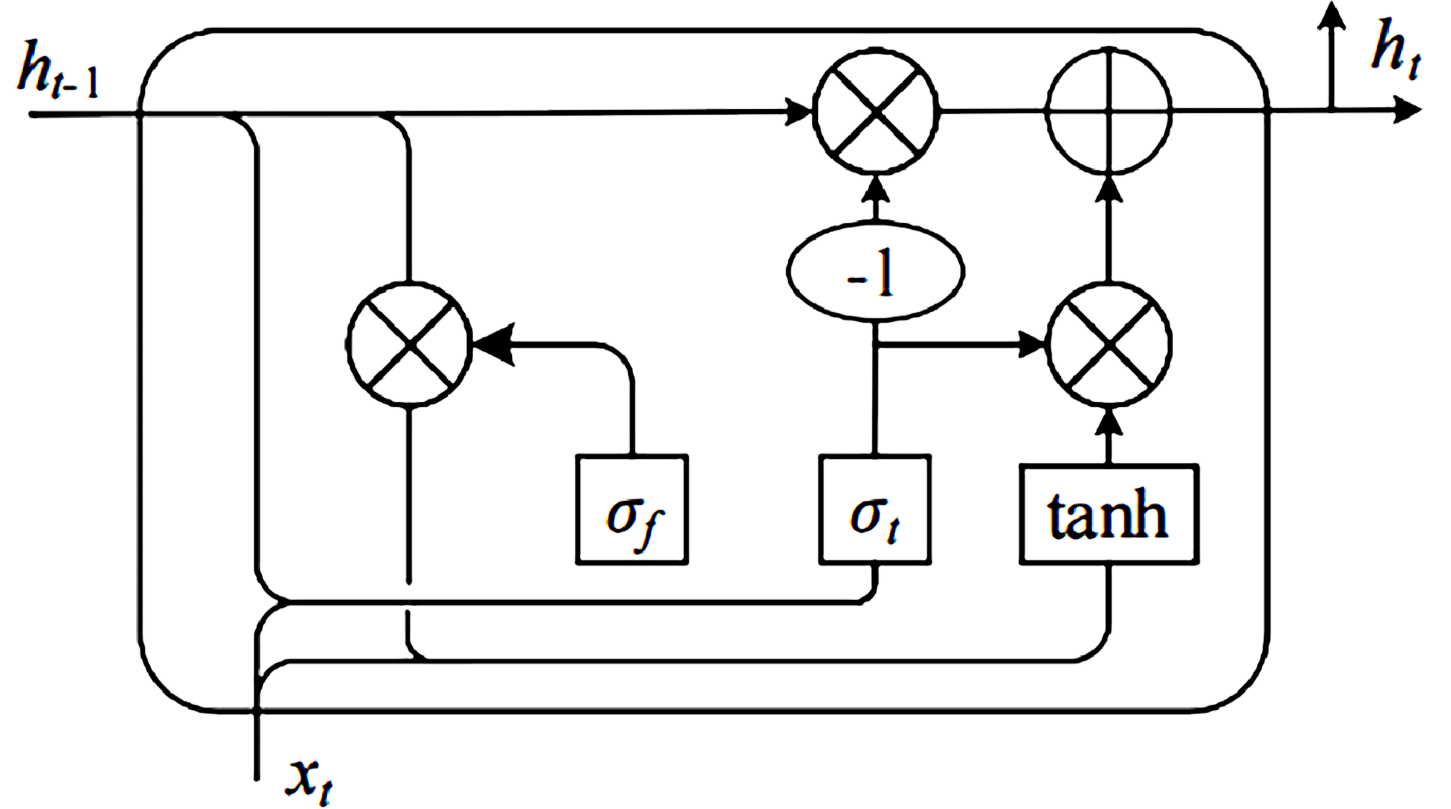
Fig Structural diagram of GRU.

The GRU maintains the same effect as the LSTM and combines the input gate and the forget gate in LSTM into one gate, called the update gate, so the algorithm only consists of the reset gate and the update gate. Figure 2 shows the GRU structure. The parameters are more simplified and have better convergence. Equation (2) presents the update gate.

(2) \begin{eqnarray*}{Z}_{t}=\sigma \left( {W}_{z}{S}_{t}+{U}_{z}{H}_{t-1}+{B}_{z} \right) \end{eqnarray*}

where *W*_*z*_ and *U*_*z*_ denote weights, and *H*_*t*−1_ represents the input and *B*_*z*_ denotes the bias.

Then, GRU calculates the candidate content that needs to be retained. *W* and *U* denote weighted, and *B* represents the bias:

(3) \begin{eqnarray*}{\hat {H}}_{t}=\tanh \nolimits \left( W{S}_{t}+U{R}_{t}{H}_{t-1}+B \right) .\end{eqnarray*}

Finally, the GRU calculates the final output based on the above-obtained results:

(4) \begin{eqnarray*}{H}_{t}= \left( 1-{Z}_{t} \right) {\hat {H}}_{t}+{Z}_{t}{H}_{t-1}.\end{eqnarray*}

In the Bi-GRU layer, the emotional features are extracted in the forward and backward GRU networks, which can capture the global features of the context. The two reverse GRUs jointly determine the output of the Bi-GRU network.

### Attention layer

As one of the most substantial concepts in deep learning, the attention mechanism is a weight allocation method that simulates the allocation mechanism of human brain attention. At a particular moment, the human brain will focus on a specific area that needs attention, reducing or ignoring other regions to grasp more valuable information (Niu, Zhong & Yu, 2021). The Attention mechanism assigns more significant weight to crucial details based on probability distribution so that the algorithm can pay more attention to this type of information. In short, an attention mechanism-based text classification can amplify core keywords’ impact on text features. As shown in Fig. 1, The result of the weight distribution will affect the final classification outcome (Liu et al., 2019). Equations (5) and (6) present attention allocation.

(5) \begin{eqnarray*}{C}_{t}& =\sum _{t=1}^{{T}_{h}}\,\,{a}_{ij}{H}_{t}\end{eqnarray*}

(6) \begin{eqnarray*}{a}_{ij}& =\text{softmax} \left( {W}_{a2}\tanh \nolimits \left( {W}_{a1}{H}_{t} \right) \right) \end{eqnarray*}

where *a*_*ij*_ denotes attention weight, *H*_*t*_ represents the length of the data, *W*_*a*1_ and *W*_*a*2_ designate weights.

## Experimentation

In this section, experiments are conducted to evaluate the performance of the sentiment classification algorithm proposed, and the educational review dataset is implemented in the article.

### Experimental procedure

• **Data collection and pre-processing:** First, we utilized crawler technology to collect the comments written by ten high school mathematics courses that high school students participated in on the Bilibili platform. The total number of texts is 20,000 in Chinese. Furthermore, the collected corpus is pre-processed to remove irrelevant comments, meaningless URLs, and non-Chinese comments (including comments with only symbols and English characters). A total of 12,655 usable comments are obtained.
• **Labeling:** The comments with obvious positive adjectives and adverbs are marked as positive, sentences without adjectives are marked as unfavorable, and adjectives or adverbs with negative emotional color and interrogative sentences are marked as negative. Through iterative processing, 6,929 positive text comments, 3,686 neutral comments, and 2,042 negative comments were obtained. Since the number of features has a particular impact on the precision analysis, “more is better” does not guarantee better outcomes. Therefore, the feature selection process must consider both efficiency and precision (Zheng, Wang & Gao, 2018). Therefore, 2,500 positive reviews were selected randomly. The distribution of the data set is shown in Table 1.
• **Word representation:** We use the trained Chinese model to vectorize Chinese text comments, and the processed text vector has 768 dimensions.
• **Building deep sentiment classification model:** Al-BERT-Bi-GRU-Att+LDA: The bi-GRU model with attention mechanism based on the proposed Al-BERT pretraining algorithm. In the Bi-GRU layer, the input dimension is consistent with the dimensions of the Al-BERT layer, and the hidden layer dimension is assigned to 256. The input sentence length is assigned to 100. In the attention layer, different weights are assigned to each word through the attention mechanism, and topic clustering is performed on the comments classified as negative.
• **Optimizing model:** During the training stage, the hyperparameters are modified to optimize the final model’s performance.
• **Model evaluation:** We evaluate the prediction results with precision, recall, and F1 score.
• **Topic clustering** After the model obtains the final classification result; the LDA topic model is used for topic clustering for the comments whose classification results are negative. Thus, the problems that learners are concerned about can be detected and the course developers can present solutions.

### Evaluation criteria

Evaluating the model performance is an essential link, and different evaluation indicators comprehensively reflect the quality of the model’s performance. The model evaluation indicators used in the article are precision, precision, recall, and the F1-scores, consistent with most studies’ indicators. They are presented in Table 2.

(7) \begin{eqnarray*}precision& = \frac{TP+TN}{TP+TN+FP+FN} \end{eqnarray*}

(8) \begin{eqnarray*}\text{Precision}& = \frac{TP}{TP+FP} \end{eqnarray*}

(9) \begin{eqnarray*}\text{Recall}& = \frac{TP}{TP+FN} \end{eqnarray*}

(10) \begin{eqnarray*}F1& = \frac{2\times Precision\times Recall}{\text{Precision}+\text{Recall}} .\end{eqnarray*}

**Table 1:** Dataset statistics.

Dataset	Positive	Neutral	Negative	Total
Train set	4,170	2,288	1,274	7,732
Validation set	1,205	602	345	2,152
Test set	1,471	766	416	2,653

### Parameter setting

The experiment’s data set was 5-fold cross-validated to obtain accurate experimental results, and the samples were randomly divided into five groups. One group was used for training, and the remaining four were used for the validation and test sets. The research compares the performance of LSTM, Bi-LSTM, Bi-LSTM-Att, Text-CNN, and Al-BERT-Bi-GRU-Att algorithms regarding precision and loss value, respectively. The hyperparameters of the proposed algorithm are shown in Table 3:

### Baseline methods

To verify the actual performance of the proposed algorithm, baseline text classification models are implemented as controls.

LSTM: The input dimension is assigned to 300, and the number of hidden layer dimensions is 300.

Bi-LSTM: the input dimension is word vector size, and the number of hidden layer dimensions is set to 300

Bi-LSTM-Att: The hidden layer dimension is assigned to 300 and combined with an attention mechanism to achieve accurate weight allocation.

Text-CNN: The filter length is 300, and the widths are set to 3,4 and 5, respectively.

**Table 2:** Classification results of the model.

True situation	Forecast result	
	Positive	Negative
Positive	TP	FN
Negative	FP	TN

**Table 3:** Parameter list.

Parameter name	Parameter value
Word vector	AlBERT
Vector dimension	768
Loss function	Cross entropy
Optimizer	Adam
Maximum text length	512
Batch size	64
Epoch	150

### Findings

#### Model comparison

**Figure 3:**
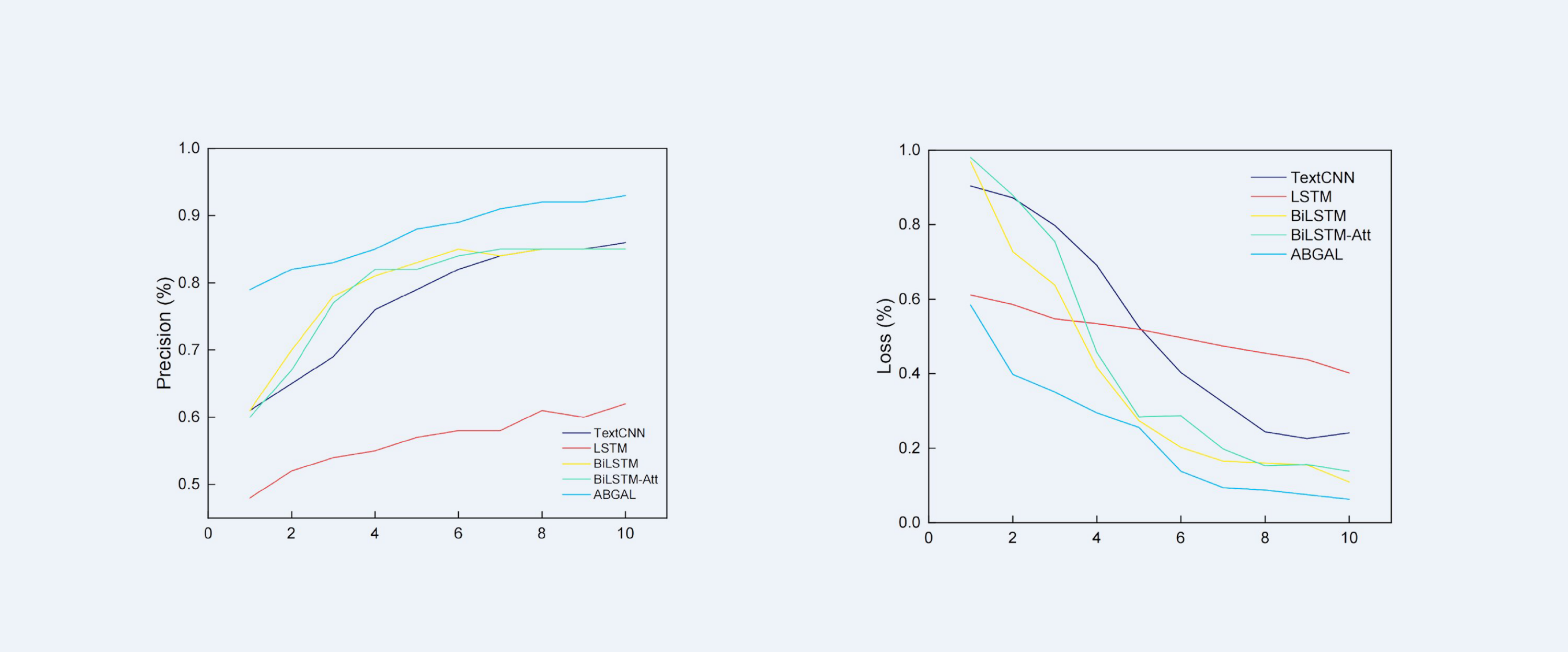
Precision and loss rate of different deep learning models.

The precision and loss values of the algorithm on the dataset are shown in Fig. 3. According to the precision rate curve, the precision rate of LSTM is lower than that of other classification algorithms. By the 10th training cycle, the precision rate has just reached 0.62 in the first training cycle of other algorithms. Text-CNN reached the highest precision rate of 0.86 in the 10th training cycle, and then the precision rate stabilized at 0.86 and fluctuated back and forth. The overall change in the precision of Bi-LSTM and Bi-LSTM-Att is relatively small, reaching the same level in the tenth training cycle. Both are 0.85, which is slightly lower than the Text-CNN model. Compared with the baseline algorithm, the proposed algorithm reaches the highest level of 0.93 in the 10th training cycle. It employs Al-BERT as the pretraining model, which reduces the risk of overfitting and the computational overhead, which is better than the baseline algorithm.

 According to the loss rate curve, the loss value of LSTM is the largest among all deep learning algorithms. Compared with the LSTM algorithms, the loss rate of Bi-LSTM decreases significantly, and it has better convergence. When compared with the Bi-LSTM algorithm, the precision and loss rates of Bi-LSTM-Att do not considerably change the precision and loss rate scores. Compared with other types of neural networks, Text-CNN performs well overall because the convolutional layer is good at extracting local features to handle classification tasks better.

The proposed model has undergone 5-fold cross-validation, and the loss value is reduced to about 6% in the final stage. Compared with other experimental algorithms, the precision of the proposed algorithm has increased by at least 5%, and the loss rate dropped by at least 3%. Experiments show that Al-BERT-Bi-GRU-Att-Softmax can enhance feature extraction capability and improve classification precision.

#### Topic clustering

When the model’s classification is a concern, we also perform topic clustering on predicted negative reviews. Hew, Qiao & Tang (2018) suggested that course reviews were divided into six topics for analysis. The primary process is to divide all negative comments into six topics for statistics after word frequency statistics are performed on the dataset: course content, course difficulty, video quality, resource acquisition, teacher attributes, and online interactions. Also, word frequency statistics for keywords under each topic are performed and quantitative analysis is conducted based on the final statistical results. Table 4 presents the outcomes.

### Discussion

According to the statistical results of negative comments under each topic, the following conclusions are attained: First, most online learners generally reflect that the course content is challenging to understand, and the verbal expressions such as “difficult,” “complex”, and “do not understand” appear the most frequently, generally reflecting that the course content is hard to understand. That is mainly related to the nature of the mathematics itself. Some students also discuss questions about mathematics knowledge in the comment area. Students generally feel anxious about learning mathematical subjects and the exams they take. Secondly, there is a lot of feedback on bullet chatting. Many students complain that the content of bullet chatting is very messy and chaotic, and most of them have nothing to do with the course content, which affects their learning success. Some students say that the video screen suddenly turned black, they could not watch it, and the video sound was low.

Moreover, some students reported that more than the video content was needed and hoped to increase the range. Students have less negative feedback about teachers regarding teacher attributes since high teaching quality is available in online courses. Participants also reported that the order of the courses needed to be corrected. Regarding resource acquisition, a few students noted that courseware and handouts could not be downloaded because links expired.

**Table 4:** Topic clustering quantitative statistical results.

Subject	Keywords	Result
Course difficulty	Difficult, Complex(9)	594
Online interaction	Bullet comment	97
Video quality	Video, Sound(3)	23
Teacher attributes	Voice, Speech rate(5)	23
Resource accessibility	link expired(3)	13

## Conclusion

The research innovation lies in the quantitative and qualitative analysis of texts when educational reviews generate texts. To do so, the combination of deep sentiment classification and topic models is conducted to derive more insights. The proposed algorithm effectively resolves the ambiguity problems in comment semantics. Experiments show that it generates a better classification result. A more than 5% precision rate is gained when compared with other deep learning algorithms that deal with special sentiment classification tasks, such as educational reviews.

Meanwhile, negative comments are employed to explore the distribution of various topics. The shortcoming of the article is that it only classifies educational review texts whose size is limited. Therefore, more research is needed to examine more extensive texts. Future work will extend this research to larger data sets and try to improve the proposed algorithm, further reviewing its effectiveness and potential impact on performance improvement.

##  Supplemental Information

10.7717/peerj-cs.2029/supp-1 Supplemental Information 1 Computer Code in Python

10.7717/peerj-cs.2029/supp-2 Supplemental Information 2 Dataset

## Additional Information and Declarations

### Data Availability

The following information was supplied regarding data availability: The code and raw data are available in the Supplemental Files.
